# Sishen Wan Treats Ulcerative Colitis in Rats by Regulating Gut Microbiota and Restoring the Treg/Th17 Balance

**DOI:** 10.1155/2022/1432816

**Published:** 2022-12-30

**Authors:** Yingyun Wang, Xiangdong Zhu, Yonglin Liang, Xinze Li, Yan Wang, Jie Li

**Affiliations:** ^1^Gansu University of Chinese Medicine, Lanzhou 730000, China; ^2^Ningxia Medical University, Yinchuan 750004, China; ^3^Tianjin University of Traditional Chinese Medicine, Tianjin 301617, China

## Abstract

**Objective:**

This study was aimed to explore the mechanism of Sishen Wan (SSW) in treating ulcerative colitis (UC) in a rat model of spleen-kidney yang deficiency pattern by regulating gut microbiota and the content of butyric acid in short-chain fatty acid (SCFAs) and restoring regulatory T (Treg)/T helper type 17 (Th17) balance from the perspective of the correlation between gut microbiota and immune function.

**Methods:**

The UC rat model of spleen-kidney yang deficiency pattern was established by the method of combining disease and syndrome (intragastric administration of senna leaf, subcutaneous injection of hydrocortisone, and enema with 2,4-dinitrobenzenesulfonic acid (DNBS)/ethanol solution). After successful modeling, rats were randomly divided into six groups: the blank group, model group, low-, middle-, and high-dose Sishen Wan groups, and mesalazine group. Samples were taken after continuous administration for 3 weeks. The general conditions and body weight of the rats were observed and recorded, and the disease activity index (DAI) score was calculated. Colonic mucosal injury was observed, and a colonic mucosal damage index (CMDI) score was calculated. Histopathological changes in colon tissues were determined by hematoxylin and eosin (H&E) staining, and the histopathological score (HS) was calculated. The serum levels of transforming growth factor-*β*1 (TGF-*β*1), interleukin (IL)-6, IL-10, and IL-17 were determined by enzyme-linked immunosorbent assay (ELISA) assays. The expression of TGF-*β*1, signal transducer and activator of transcription 3 (STAT3), and peroxisome proliferator-activated receptor *γ* (PPAR*γ*) was determined by Western blot analysis. The proportion of Th17 and Treg cells in colon tissue was determined by flow cytometry. The relative abundance of gut microbiota was determined by 16S rDNA sequencing, and the concentration of butyric acid of SCFAs was determined by gas chromatography-mass spectrometry (GC-MS).

**Results:**

Administration of SSW significantly improved the pathological changes of colon tissue in UC rats and could attenuate the DAI and CMDI scores, and the HS. SSW significantly decreased the serum levels of IL-6 and IL-17 and increased the serum levels of TGF-*β*1 and IL-10. In addition, SSW increased the expression of TGF-*β*1 and PPAR*γ* and decreased the expression of STAT3 in colon tissue in a dose-dependent manner. Furthermore, SSW significantly decreased the proportion of Th17 cells and increased the proportion of Treg cells in colon tissue. Additionally, SSW altered the gut microbiota, including an increase in the relative abundance of Firmicutes and a decrease in Bacteroidota at the phylum level and an increase in the relative abundance of *Lactobacillus* at the genus level. Moreover, SSW significantly increased the concentration of butyric acid.

**Conclusions:**

Combined, these data suggested that SSW increased the relative abundance of firmicutes and the level of butyric acid and restored the balance of Treg/Th17 immune axis and gut homeostasis, thus delaying the progress of UC.

## 1. Introduction

Ulcerative colitis (UC) is a type of inflammatory bowel disease (IBD) characterized by idiopathic chronic inflammation of the colonic mucosa, which begins in the rectum and continues to extend to the proximal colon [1]. Typical symptoms include abdominal pain, bloody diarrhea and mucus, fecal urgency, and tenesmus [2]. Patients with UC usually relapse and experience remission, and up to 90% of patients relapse one or more times after the first episode [3]. The pathogenesis of UC is unclear, but it has been confirmed to be related to immune response disorder, a change in gut microbiota, genetic susceptibility, and environmental factors [4]. In recent years, based on studying the immune mechanisms underlying chronic inflammatory diseases, there has been a deeper understanding of the pathogenesis of UC.

Regulatory T (Treg)/T helper type 17 (Th17) immune imbalance, a common inflammatory-related mechanism mediated by T cells, is considered an important mediator of tissue damage causing colonic mucosal ulceration in UC [5]. Tregs and Th17 cells are derived from CD4^+^T lymphocytes, and IL-6 plays a key role in CD4^+^T lymphocyte differentiation by activating the signal transducer and activator of transcription 3 (STAT3) to phosphorylate STAT3, thus promoting the occurrence and development of UC [6]. As important proinflammatory cells, Th17 cells promote intestinal inflammatory reactions by specifically secreting IL-17. Tregs inhibit the activity of immune cells and control inflammation by secreting transforming growth factor-*β* (TGF-*β*) and IL-10 [7–9]. The gut microbiota of UC patients is related to the Treg/Th17 immune axis. In previous studies, it has been shown that the Treg/Th17 balance in intestinal mucosa can be improved by increasing the production of microbial short-chain fatty acids (SCFAs), so as to prevent and treat IBD [10]. SCFAs are metabolites formed by intestinal microbial glycolysis of dietary fiber, mainly including acetic acid, propionic acid, and butyric acid in which butyric acid can be used as an agonist of peroxisome proliferator-activated receptor *γ* (PPAR*γ*) [11, 12]. PPAR*γ* can restore the pathophysiological imbalance associated with IBD by inhibiting the production of inflammatory cytokines, thereby improving the impaired intestinal barrier function under the inflammatory state [13]. Moreover, PPAR*γ* plays an important regulatory role in Th17 and Treg differentiation [14].

Drugs that are commonly used in the clinic to treat UC include 5-aminosalicylic acids, steroids, and immunosuppressants [15, 16]. However, these drugs often lead to different side effects, such as nephrotoxicity, and increase the risk of infection and surgical complications, thereby reducing the quality of life of patients, especially during long-term treatment [17]. Traditional Chinese medicine (TCM) plays an auxiliary role in the treatment of UC, and its popularity is increasing worldwide [18]. However, the active components and exact underlying mechanism of action of TCM are still unclear. UC is divided into seven types of TCM, of which the spleen-kidney yang deficiency pattern is one of the most important types [19]. The main clinical manifestations of UC with a spleen-kidney yang deficiency pattern are fear of cold, soreness and weakness of the waist and knees, mental fatigue, abdominal distension, and loose stool [20]. Sishen Wan (SSW) is the main prescription for treating UC of the spleen-kidney yang deficiency pattern, and it is also the classic prescription in TCM for treating colitis [19,21]. Increasing data from evidence-based medicine indicate that SSW has a good therapeutic effect on IBD, and the total effective rate of 204 patients with IBD is 75.98% [22]. At present, there are relatively few studies on the single prescription of SSW, and most of them tend to be clinical studies, with few studies on the mechanism of action of SSW. Gut microbiota imbalance and immune disorders are the main etiology and pathogenesis of UC, but the mechanism of SSW in the treatment of UC by regulating gut microbiota and restoring the Treg/Th17 balance is not completely clear. In this study, the UC rat model with a spleen-kidney yang deficiency pattern was established by the method of combining disease and syndrome, and the mechanism of SSW on UC was verified by animal experiment.

## 2. Materials and Methods

### 2.1. Materials and Reagents

2,4-Dinitrobenzenesulfonic acid hydrate (DNBS) was purchased from Sigma (Germany). Hydrocortisone injection was purchased from Enterprise Group Rongsheng Pharmaceutical Co., Ltd. (China). Senna leaf was purchased from Huirentang pharmacy (China). Hematoxylin and eosin were purchased from Beijing Solarbio Science & Technology Co., Ltd. (China). ELISA kits to detect IL-6, IL-10, IL-17, and TGF-*β*1 were purchased from Jiangsu Feiya Biotechnology Co., Ltd. (China). Rabbit antibodies directed against TGF-*β*1, PPAR*γ*, and STAT3 were purchased from Abcam (UK). A rabbit antibody directed against p-STAT3 was purchased from Cell Signaling Technology (USA). Goat anti-rabbit immunoglobulin and goat anti-mouse immunoglobulin were purchased from Immunoway (USA). Anti-Mouse CD25, FOXP3, and Th17 were purchased from Multi Sciences (Lianke) Biotech Co., Ltd. (China).

### 2.2. Animals

A total of 120 Sprague-Dawley (SD) rats (10 weeks of age, 200 ± 20 g, males and females) were purchased from the Experimental Animal Center of Gansu University of Chinese Medicine (Gansu, China) and attached with a health and safety certificate of conformity administered by the Chinese government (Batch number of production license is SCXK2020-0001). Rats were housed in a specific-pathogen-free (SPF) barrier laboratory of the Experimental Animal Center of Gansu University of Chinese Medicine, with a temperature that was maintained between 21–25°C and a relative humidity within 50%–60%, 12 h light/12 h dark cycle, and noise <50 dB. Rats were freely fed with standard feed and water.

### 2.3. Preparation of the Animal Model [20, 23]

The UC rat model of spleen-kidney yang deficiency pattern was established by the method of combining disease and syndrome, enema with DNBS and ethanol solution, subcutaneous injection of hydrocortisone, and intragastric administration of senna leaf. All rats were adaptively fed for 7 days before modeling. Rats (11 weeks of age) were given an intragastric injection of senna leaf (10 mL/kg) combined with a subcutaneous injection of hydrocortisone (15 mg/kg) once a day for 21 days. At 21 days later, rats were fasted for 24 h and had free access to water. Then, rats were anesthetized with 2% pentobarbital sodium (0.2 mL/100 g), and polypropylene tubes were slowly passed through the anus of each rat to a depth of about 8 cm. Next, a syringe was used to quickly inject DNBS and an ethanol solution (100 mg/kg DNBS+1 mL/kg 50% ethanol), and then about 0.4 mL of air was injected. The anus was pinched tightly, and rats were held upside down for 1 min.

General conditions of the rats, such as mental state, activity, fur, stool properties, bleeding, and body weight, were observed and recorded, and the disease activity index (DAI) score was calculated. Damage to the colonic mucosa was observed, and the colonic mucosal damage index (CMDI) score was calculated. Histopathological changes in colon tissues were detected using light microscopy, and the histopathological score (HS) was calculated. To sum up, the rat model of spleen-kidney yang deficiency pattern was successfully established [23].

### 2.4. Preparation of SSW

The dry Chinese herbal medicines, which include *Psoraleae Fructus* (the dried fruits of *Psoralea corylifolia*), *Evodiae Fructus* (the dried fruits of *Evodia rutaecarpa*), *Myristicae Semen* (the ripe seeds of *Myristica fragrants* Houtt), *Schisandrae Chinensis Fructus* (the dried fruits of *Schisandra chinensis* (Turcz.) Baill), *Zingiber Officinale Roscoe* (the rhizomes of *Zingiber officinale* Rosc.), *and Jujubae Fructus* (the ripe fruits of *Ziziphus jujuba* Mill.) were purchased from Huirentang pharmacy (Lanzhou, China, Production dates were 2020). The above-mentioned six herbs (in a ratio of 4 : 1 : 2 : 2 : 2 : 2 : 2) were mixed and immersed in 8 times distilled water for 30 min, decocted for 20–30 minutes, the residue was removed and filtered, and the herbs were then decocted twice with water for 15–20 min. Three batches of filtrates were mixed. The filtrates were concentrated to a low dose of crude drug content (0.6 g/mL), a middle dose of crude drug content (1.2 g/mL), and a high dose of crude drug content (2.4 g/mL). Samples were stored in the dark at 4°C.

### 2.5. Grouping and Administration of the Animal Model

After successful modeling, rats were randomly divided into six groups (20 rats per group): the blank group, the model group, the low-dose group (SSW-L, 6 g/kg of SSW), middle-dose group (SSW-M, 12 g/kg of SSW), the high-dose group (SSW-H, 24 g/kg of SSW), and the mesalazine group (mesalazine, 0.36 g/kg) [23]. The administered doses were converted based on the body surface areas of humans and rats. The blank group and model group were given an equal volume of distilled water (10 mL/kg), and each group received an intragastric administration once a day for 21 consecutive days.

### 2.6. Collection of Specimens

After treatment, rat fecal samples were collected by the abdominal massage method and used for detection by 16S rDNA sequencing and chromatography-mass spectrometry (GC-MS). Rats were fasted for 24 h then anesthetized with 2% pentobarbital sodium (0.2 mL/100 g). Blood samples were collected from the abdominal aorta and centrifuged to obtain serum, which was used for enzyme-linked immunosorbent assay (ELISA). Then the rats were euthanized. Colon samples were divided into three segments: (1) One segment was fixed with 4% paraformaldehyde for hematoxylin and eosin (H&E) staining for histological observation. (2) One segment was placed in physiological saline and immediately used for flow cytometry. (3) The last segment was stored at −80°C for Western blot analysis.

### 2.7. General Condition of Rats

Mental state, activity, fur, stool property, bleeding, and body weight of rats were monitored and recorded daily. According to the literature [24], the following classification method was used: (a) body weight loss: 0 = less than 1%; 1 = 1–5%; 2 = 6–10%; 3 = 11–15%; 4 = over 15%; (b) stool property: 0 = normal; 1 = soft but still formed; 2 = soft; 3 = very soft and wet; 4 = watery diarrhea; (c) blood in stool: 0 = normal (-); 1 = negative hemocult (+); 2 = positive hemocult (++); 3 = blood traces in stool visible (+++); 4 = gross bleeding (++++), the DAI score was calculated, and the mean of all scores to quantify the deterioration of UC was recorded.

### 2.8. Histopathology

Fresh colon samples were washed in physiological saline to remove the surrounding connective tissue and fat. Colonic mucosal injury was observed with the naked eye, and the CMDI score was calculated. The CMDI score was calculated based on the following classifications: (a) 0 = no damage; (b) 1 = mild hyperemia, oedema, smooth surface, no erosion damage; (c) 2 = moderate hyperemia, oedema, coarse granular mucosa, erosion, or intestinal adhesions; (d) 3 = highly congestive and edematous, with necrosis and ulcer formation on the mucosal surface, maximum longitudinal diameter of the ulcer <1 cm, thickening of the intestinal wall, or necrosis and inflammation on the surface; (e) 4 = maximum longitudinal diameter of the ulcer >1 cm on a 3-point basis or total necrosis of the bowel wall [25].

Colon tissue was fixed with 4% paraformaldehyde, dehydrated, and embedded in paraffin. Histological evaluation of colon sections was performed by H&E staining. Scoring was done according to the literature [26], (a) 0 = normal; (b) 1 = loss of 1/3 of the crypt glands; (c) 2 = loss of 2/3 of the crypt glands; (d) 3 = loss of all the crypt glands and complete mucosal epithelium with mild signs of inflammatory cell infiltration; (e) 4 = erosion and destruction of the mucosal epithelium with obvious inflammatory cell infiltration, the tissue sections were scored for HS.

### 2.9. Determination of Cytokines

According to the manufacturer's instructions, serum levels of interleukin (IL)-6, IL-10, IL-17, and TGF-*β*1 were determined using corresponding ELISA kits. According to the instructions for the procedure, the concentration of cytokines in the serum was calculated by using a standard curve. Results were expressed as ng/L of serum per sample.

### 2.10. Western Blot Analysis

According to the standard scheme, the total protein samples were extracted from colon tissue using lysis buffer. The protein concentration in the extracts was quantified by the BCA method. Samples were loaded on a gel and then transferred to polyvinylidene fluoride (PVDF) members. After blocking, membranes were incubated overnight with primary antibodies directed against *β*-actin (Servicebio, Lot: LS193437, 1 : 2000 dilution, Wuhan, China), STAT3 (Abcam, ab68153, 1 : 2000 dilution, UK), TGF-*β*1 (Abcam, ab215715, 1 : 1000 dilution, UK), PPAR*γ* (Abcam, ab209350, 1 : 1000 dilution, UK), and p-STAT3 (Cell Signaling Technology, 9145S, 1 : 2000, USA) at 4°C, and subsequently incubated with secondary antibodies, including goat anti-rabbit immunoglobulin (1 : 8000) and goat anti-mouse immunoglobulin (1 : 8000) for 2 h at room temperature. Finally, protein bands were visualized with chemiluminescence reagents and observed with a gel imaging system. The bands were analyzed by Image J software.

### 2.11. Flow Cytometry

Fresh colon tissues from rats were placed on a superclean bench to remove the surrounding connective tissues. A proper amount of sterilized PBS was added, and the mixture was fully ground and filtered to prepare a single-cell suspension. The cells were resuspended in 100 *μ*L of PBS containing fetal calf serum, and 2-Acetoxy-1-methoxypropane (PMA) (1 *μ*g/mL), ionomycin (50 *μ*g/mL), and monamycin (0.1 mg/mL). Cells were stimulated and cultured in an incubator for 6 hours (37°C, 5% CO_2_). After breaking the membrane and fixation, an appropriate amount of Th17 antibody was added, according to the instructions, and the percentage of Th17 cells was determined by flow cytometry. An appropriate amount of CD25 and Foxp3 antibodies were added, and the percentage of Treg cells was determined by flow cytometry.

### 2.12. 16S rDNA Gene Microbiome Analysis

Total genome DNA from samples was extracted using the CTAB/SDS method. The 16S rRNA gene hypervariable V3-V4 region was amplified using specific primers with the barcode. The prototype primers were 515F (CCTAYGGGRBGCASCAG)-806R (GGACTACNNGGGTATCTAAT). All PCR reactions were carried out in 15 *μ*L of Phusion® High-Fidelity PCR Master Mix (New England Biolabs); 0.2 *μ*M of forward and reverse primers, and 10 ng of template DNA. The same volume of 1X loading buffer (contained SYB green) was mixed with PCR products, and electrophoresis was performed on 2% agarose gels. Sequencing libraries were generated using the TruSeq® DNA PCR-Free Sample Preparation Kit (Illumina, USA) and index codes were added. The library's quality was assessed on the Qubit@ 2.0 Fluorometer (Thermo Scientific) and an Agilent Bioanalyzer 2100 system. Finally, the library was sequenced on an Illumina NovaSeq platform, and 250 bp paired-end reads were generated. Sequences with ≥97% similarity were assigned to the same operational taxonomic units (OTUs). The representative sequence for each OTU was screened for further annotation.

### 2.13. Measurement of Short-Chain Fatty Acids

The level of short-chain fatty acid was measured by GC-MS analysis. A total of 50 *μ*L 15% phosphoric acid, 100 *μ*L isohexic acid solution, and 400 *μ*L diethyl ether were added to each sample, grind, centrifuged, and the supernatant was tested. The obtained SCFAs samples were measured using a TRACE 1310-ISQ LT GC–MS system (Thermo, USA). Based on the detection results, target quantification was carried out on the detected samples, and relevant data analysis was carried out.

### 2.14. Statistical Analyses

SPSS 23.0 was used for statistical analysis, and one-way ANOVA was used for data analysis. The least significant difference test (LSD) was used for comparison between groups with homogeneity of variance, and the Tamhane's T2 test was used for non-homogeneity of variance. All results were expressed as the mean ± SD and *P* < 0.05 was considered statistically significant.

## 3. Results

### 3.1. SSW Improves the General Condition of Rats

Rats in the blank group were in a good mental state, and the action response was very flexible. In addition, the rat fur was smooth and lustrous, the stool was normal, and the body weight increased steadily. After modeling, rats showed a reduced food intake, mental malaise, decreased activity, slow reactions, a fondness for clustering, loose stools, and damp and dirty abdominal fur. After induction of enema with DNBS and ethanol solution, rats showed obvious bloody stool and a dirty perianal region. The body weight of rats decreased at the late modeling stage. After treatment, compared with the model group, the symptoms of rats in the SSW-L group were slightly improved; the manifestations included slightly increased food intake, a poor mental state, less activity, a fondness for clustering , still-present lack of luster in fur color, and loose and shapeless stool. The symptoms of rats in the SSW-M, SSW-H, and mesalazine groups were significantly improved with increased food intake, a better mental state, increased activity, improved huddle, restored luster in fur color, formed stool, and a gradually cleaned perianal region. The body weight of rats in the model group did not change much during modeling (Table 1). The body weight of rats after treatment is presented in Table 2. Compared with the model group, the body weight of rats in the SSW-M, SSW-H, and mesalazine groups increased significantly (*P* < 0.05, *P* < 0.01).

Compared with the blank group, the DAI score of rats in the model group was significantly increased at the end of modeling (*P* < 0.01). After drug treatment, compared with the model group, the DAI score of rats in each treatment group was significantly decrease (*P* < 0.01). No significant differences were observed between rats in the mesalazine and SSW-H groups (Table 3).

### 3.2. SSW Improves Colonic Tissue Injury

**Figure 1:**
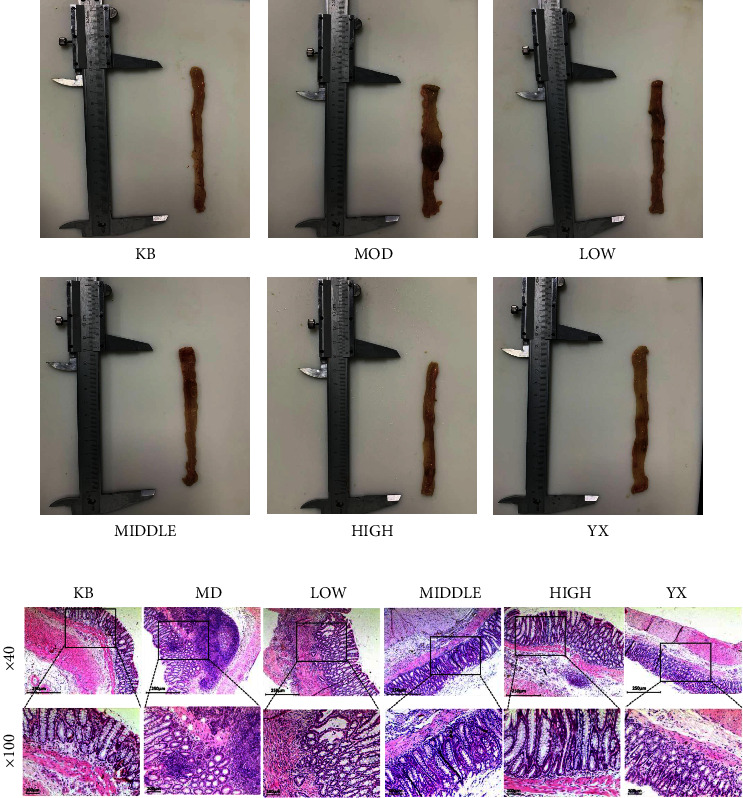
(a) Gross appearances of the colon in the six treatment groups. KB: blank group; MOD: model group; LOW: SSW-L group; MIDDLE: SSW-M group; HIGH: SSW-H group; YX: mesalazine group. (b) Pathological changes in colon tissues of rats in each group (magnification of ×40 and ×100, scale bar = 250 *μ*m and 100 *μ*m). KB: blank group; MOD: model group; LOW: SSW-L group; MIDDLE: SSW-M group; HIGH: SSW-H group; YX: mesalazine group.

The appearance of the colon was evaluated as shown in Figure 1(a). The gross morphology of colonic tissue was observed with the naked eye. In the blank group, the intestinal mucosa of rats was smooth, without congestion, ulcers, or other abnormal manifestations. Compared with the blank group, rats in the model group had thickened and hypertrophic intestines, congestion, edema, and erosion of intestinal mucosa, obvious ulcers on the surface, and severe adhesion or necrosis of some part of the intestines with surrounding organs. Compared with the model group, the intestinal wall of rats in the SSW-L group was thickened, and congestion and edema were still observed in the intestinal mucosa without a large ulcer surface, and there was occasional adhesion and necrosis between the intestines and surrounding organs. Rats in the SSW-M group, SSW-H group, and mesalazine group showed remarkable recovery of intestinal mucosal injury, and ulcers were rarely observed. A small number of rats still showed mild congestion and edema, but there was no adhesion with surrounding organs. As shown in Table 4, compared with the blank group, the CMDI score of rats in the model group was significantly increased. Compared with the model group, the CMDI score of rats in all treatment groups was significantly reduced (*P* < 0.01). No significant differences were observed between rats in the mesalazine group and SSW-H group.

The results of H&E staining showed that in the blank group, the colonic tissues were stained evenly, with each structure clearly visible, the intestinal mucosa was arranged neatly, and glands were distributed evenly. Compared with the blank group, the colon tissue in the model group was seriously damaged, and the intestinal mucosa disappeared. The glandular structure was damaged, and many inflammatory cells were observed. Compared with the model group, the degree of damage to colon tissue, the intestinal mucosa, and glandular structure in the SSW groups were reduced in a dose-dependent manner, and the number of inflammatory cells was reduced in a dose-dependent manner. The effects in the SSW-H and mesalazine groups were the best (Figure 1(b)). Compared with the blank group, the HS score of rats in the model group was significantly increased (*P* < 0.01). Compared with the model group, the HS scores of all treatment groups were significantly decreased (*P* < 0.01). No significant differences were observed between rats in the mesalazine group and SSW-H group (Table 5).

### 3.3. SSW Regulates the Level of Cytokines in the Colon of UC Rats

**Figure 2:**
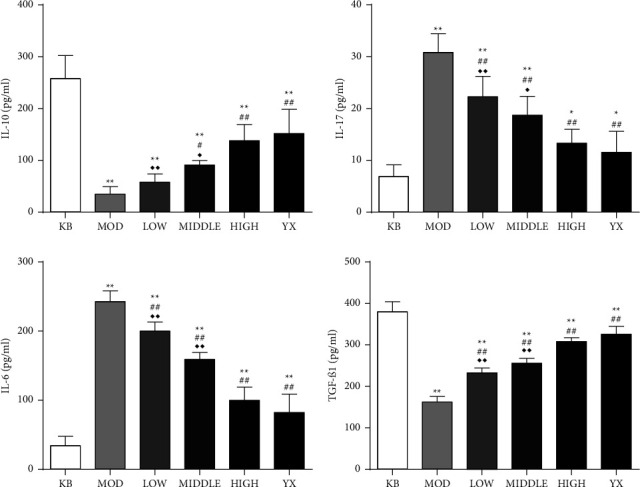
SSW changes the content of inflammatory factors in colon tissue of UC rats. KB: blank group; MOD: model group; LOW: SSW-L group; MIDDLE: SSW-M group; HIGH: SSW-H group; YX: mesalazine group. ^*∗∗*^*P* < 0.01 compared with the blank group; ^##^*P* < 0.01, ^#^*P* < 0.05 compared with the model group; ^◆◆^*P* < 0.01,^ ◆^*P* < 0.05 compared with the mesalazine group (mean ± SD, (*n* = 8).

By detecting the levels of related inflammatory factors in colon tissue of rats with UC, compared with the blank group, the levels of IL-6 and IL-17 were significantly increased, and the levels of IL-10 and TGF-*β*1 were significantly decreased (*P* < 0.01) in the model group. Moreover, compared with the model group, the levels of IL-6 and IL-17 were significantly decreased (*P* < 0.01), and the levels of IL-10 and TGF-*β*1 were increased in the SSW groups in a dose-dependent manner. No significant differences were observed between rats in the mesalazine group and SSW-H group (Table 6, Figure 2).

### 3.4. SSW Restores the Balance of Th17/Treg in UC Rats

**Figure 3:**
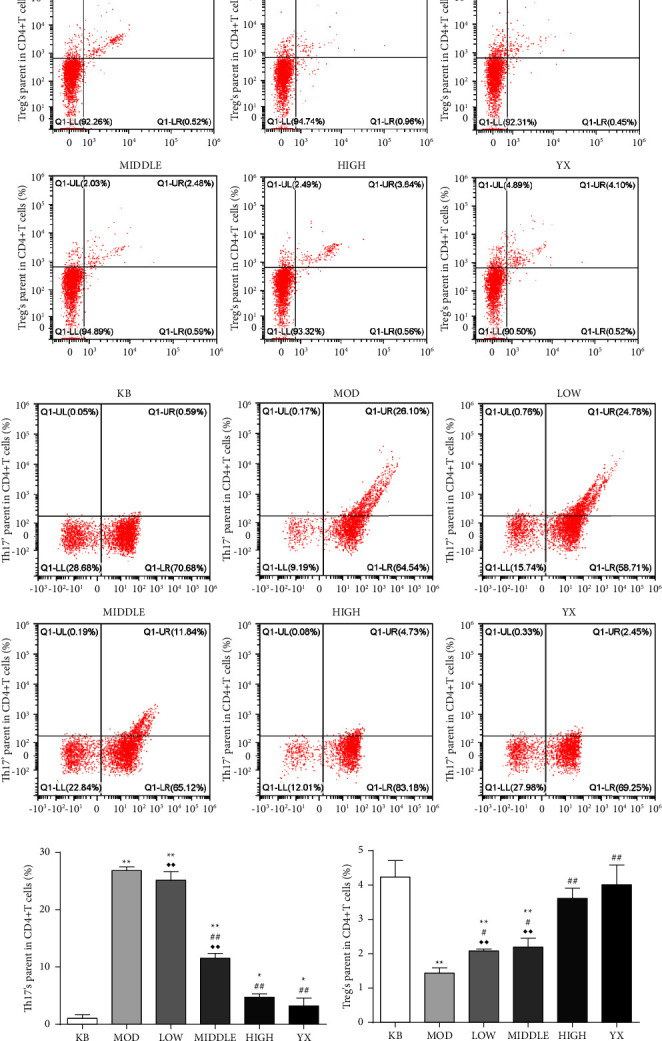
SSW restores the balance of Treg/Th17 immune axis in the colon tissue of UC rats. (a) Representative image of the proportion of Treg cells. (b) Representative image of the proportion of Th17 cells. (c) Changes in the proportion of treg cells and Th17 cells. KB: blank group; MOD: model group; LOW: SSW-L group; MIDDLE: SSW-M group; HIGH: SSW-H group; YX: mesalazine group. ^*∗∗*^*P* < 0.01,  ^*∗*^*P* < 0.05 compared with the blank group; ^##^*P* < 0.01, ^#^*P* < 0.05 compared with the model group; ^◆◆^*P* < 0.01 compared with the mesalazine group (mean ± SD, (*n* = 8).

To investigate the effect of SSW on the Treg/Th17 immune axis in rats with UC, we determined the proportion of Treg and Th17 cells in colon tissue by flow cytometry. Compared with the blank group, the proportion of Th17 cells in colon tissue of rats in the model group was significantly increased (*P* < 0.01), and the proportion of Treg cells was significantly decreased (*P* < 0.01). In addition, compared with the model group, the proportion of Treg cells in the colon tissue of rats in each treatment group was significantly increased (*P* < 0.01, *P* < 0.05), and the proportion of Th17 cells was significantly decreased (*P* < 0.01) in a dose-dependent manner. No significant differences were observed between rats in the SSW-H group and mesalazine group (Figure 3)

### 3.5. SSW Regulates Protein Expression in Colon Tissue of UC Rats

**Figure 4:**
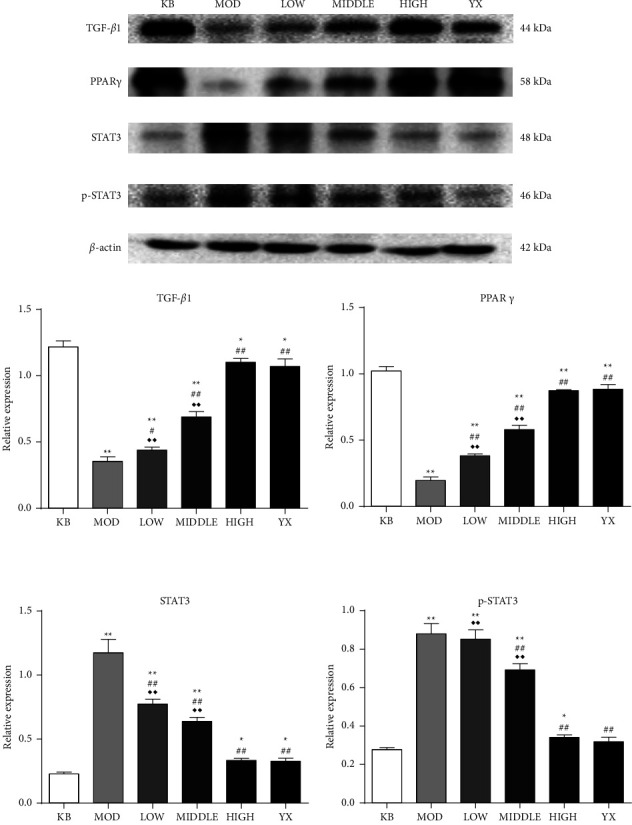
SSW changes the expression of related proteins in colon tissues of UC rats. (a) Representative images of western blot analysis. (b) The relative protein levels of TGF-*β*, PPAR*γ*, STAT3, and p-STAT3 in colon tissue. Data were normalized to the intensity of *β*-actin. KB: blank group; MOD: model group; LOW: SSW-L group; MIDDLE: SSW-M group; HIGH: SSW-H group; YX: mesalazine group. ^*∗∗*^*P* < 0.01,  ^*∗*^*P* < 0.05 compared with the blank group; ^##^*P* < 0.01, ^#^*P* < 0.05 compared with the model group; ^◆◆^*P* < 0.01 compared with the mesalazine group (mean ± SD, (*n* = 8).

Compared with the blank group, the expression of TGF-*β*1 and PPAR*γ* was significantly decreased, and STAT3 and p-STAT3 were significantly increased (*P* < 0.01) in the model group. Furthermore, compared with the model group, the expression of TGF-*β*1 and PPAR*γ* in each treatment group was significantly increased (*P* < 0.05, *P* < 0.01), the expression of STAT3 was significantly decreased (*P* < 0.01), and the expression of p-STAT3 was significantly decreased (*P* < 0.01) in the SSW-M, SSW-H, and mesalazine group. No significant differences were observed between rats in the SSW-H group and mesalazine group (Figure 4).

### 3.6. SSW Contributes to Restore the Gut Homeostasis of UC Rats

**Figure 5:**
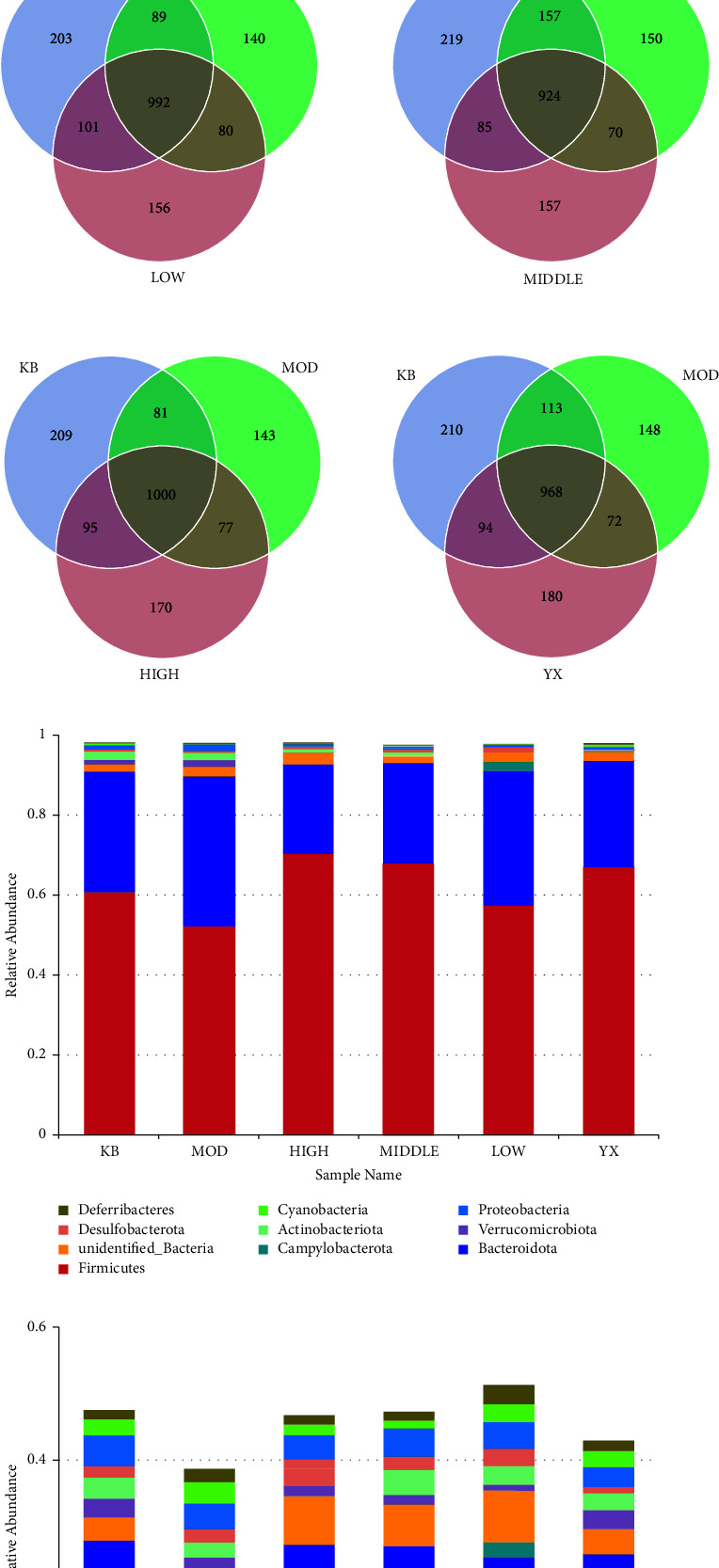
SSW improves the diversity and abundance of gut microbiota in rats. (a) Representative images of OTU. (b) Relative abundance of gut microbiota at the phylum level. (c) Relative abundance of gut microbiota at the genus level. KB: blank group; MOD: model group; LOW: SSW-L group; MIDDLE: SSW-M group; HIGH: SSW-H group; YX: mesalazine group.

Compared with the blank group, the abundance of OTU was decreased in the model group in UC rats. After drug treatment, the abundance of OTU in each treatment group was increased (Figure 5(a)). Phylum level analysis showed that compared with the blank group, the relative abundance of Firmicutes in the model group was decreased, and the relative abundance of Bacteroidota was increased, which were statistically significant (*P* < 0.05, *P* < 0.01). Compared with the model group, the relative abundance of firmicutes in all treatment groups was increased, and that of the SSW-H and mesalazine group was significantly increased (*P* < 0.01) and higher than that of the blank group. The relative abundance of Bacteroidota was decreased, and that of SSW-M, SSW-H, and mesalazine groups was decreased significantly (*P* < 0.01). The relative abundance was lower than that of the blank group (Table 7, Figure 5(b)). Genus-level analysis showed that, compared with the blank group, the relative abundance of *Lactobacillus* in the model group was significantly decreased (*P* < 0.01). Moreover, compared with the model group, the relative abundance of *Lactobacillus* in all treatment groups was significantly increased (*P* < 0.05, *P* < 0.01) (Table 8, Figure 5(c)). Taken together, these data showed that SSW increased the abundance of Firmicutes, especially the abundance of *Lactobacillus*, thereby suggesting that SSW can regulate the balance of the intestinal environment.

### 3.7. SSW Promotes Butyric Acid Production by Increasing the Abundance of Lactobacillus in Firmicutes

**Figure 6:**
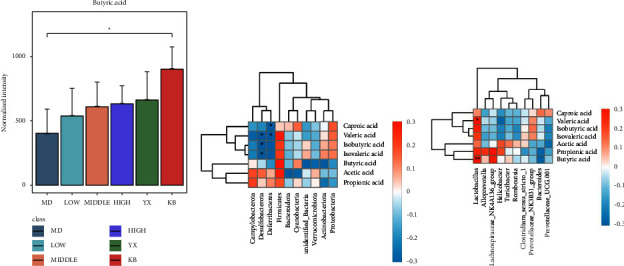
Butyric acid content and correlation between butyric acid and gut microbiota. (a) Quantitative analysis of butyric acid in each group, in *μ*g/g. (b) Association analysis of SCFAs with gut microbiota phylum levels. (c) Association analysis of SCFAs with gut microbiota genus levels. Red represents a positive correlation, and blue represents a negative correlation. KB: blank group; MOD: model group; LOW: SSW-L group; MIDDLE: SSW-M group; HIGH: SSW-H group; YX: mesalazine group.

Compared with the blank group, the butyric acid concentration was significantly decreased (*P* < 0.01) in the model group. Moreover, compared with the model group, the butyric acid concentration increased in each treatment group, and significantly increased in the SSW-H and mesalazine group (*P* < 0.05) (Table 9, Figure 6(a)). The results of correlation analysis showed that, of the phylum level, the higher the relative abundance of Firmicutes, the lower the relative abundance of Bacteroidota, and the higher the butyric acid concentration. At the genus level, the higher the relative abundance of *Lactobacillus*, the higher the butyric acid concentration (Figures 6(b) and 6(c)). These findings prove that increased firmicutes and *Lactobacillus* abundance promote the production of butyric acid.

## 4. Discussion

UC is a chronic type of IBD of unknown etiology in which the immune response plays an important role [1, 27]. According to the long course of the disease and the tendency for repeated attacks, UC is classified in TCM into the categories of dysentery and diarrhea in TCM. The basic pathogenesis of UC is spleen deficiency, and UC will develop from the spleen to the kidney over time, resulting in spleen-kidney yang deficiency.

SSW is the main prescription for the treatment of UC of the spleen-kidney yang deficiency, and is composed of *Psoraleae Fructus*, *Evodiae Fructus*, *Myristicae Semen*, *Schisandrae Chinensis Fructus*, *Zingiber Officinale Roscoe, and Jujubae Fructus*. *Psoraleae Fructus*, bitter and warm, is a sovereign drug, warming interior for dispersing cold. *Evodiae Fructus* is a minister drug, warmly invigorating spleen and stomach and relieving diarrhea with astringents. *Myristicae Semen* is also a minister drug, relieving diarrhea with astringents. *Schisandrae Chinensis Fructus* with astringent is an assistant drug. *Zingiber Officinale Roscoe* warming kidney for dispelling cold is envoy drug. *Jujubae Fructus*, which nourishes the spleen and stomach is also an envoy drug. All herbs are matched to have the effects of warming kidney and spleen and relieving diarrhea with astringents.

The active components of the six herbs are complex, and modern pharmacological action proves that the constituent drugs of SSW have an anti-inflammatory effect. The monoterpenoid compound isolated from *Psoraleae Fructus* can inhibit the expression of iNOS mRNA by inactivating nuclear factor *κ*b (NF-*κ*B) to exert an anti-inflammatory effect [28]. The water extract of *Myristicae Semen* can inhibit the expression of pro-inflammatory factors, such as IL-1*β* and IL-6 in colonic mucosa and has antidiarrheal and anti-inflammatory effects [29, 30]. Schisandrin B can up-regulate the expression of PPAR*γ*, and inhibit the activation of NF-*κ*B, thereby down-regulating the production of relevant inflammatory factors, such as IL-6 [31, 32]. *Evodiae Fructus* can significantly improve inflammatory responses in the body by regulating NLRP3 and the NF-*κ*B inflammasomes [33, 34]. The active ingredient 6-Gingerol in *Zingiber Officinale Roscoe* can prevent chronic UC by down-regulatingNF-kB and inhibiting proinflammatory cytokines and can also improve acute colitis by activating adenosine monophosphate-activated protein kinase [35–37]. Furthermore, the *Jujubae Fructus* polysaccharide can significantly inhibit the expression of proinflammatory cytokines, such as IL-6, IL-2, and plays an anti-inflammatory role [38, 39]. At present, there is no effective radical treatment for UC, and most drugs that are conventionally used for the treatment of UC will further deteriorate the intestinal microecology and promote the progression of the disease. The pharmacological mechanism of SSW in the treatment of intestinal diseases has been confirmed to some extent but only focuses on some indicators of inflammatory factors. It is not clear whether SSW can play an immune role by regulating the gut microbiota in the treatment of UC. In this study, it was confirmed that SSW can increase the content of butyric acid in the gut microbiota of UC rats, correct the imbalance of the Treg/Th17 immune axis, and play a role in alleviating inflammation and tissue damage.

The intestinal immune system protects the intestinal mucosal surface from infection and injury, and the Th17/Treg imbalance is an important characteristic of UC [40, 41]. We proposed that SSW could treat UC by regulating intestinal microbiota and restoring regulatory Treg/Th17 balance. TGF-*β* is a key regulator to maintain inflammation and immune responses [42, 43], promote mucosal healing, and protect host tissues from UC intracavitary lesions [27,44]. TGF-*β* can induce CD4^+^T cells to differentiate into Treg cells, and the presence of IL-6 can differentiate CD4^+^T cells into Th17 cells [27]. IL-10 can inhibit the production of inflammatory factors, such as IL-6 and thus inhibiting the production of Th17 cells by reducing the antigen-presenting ability of monocytes [27]. Th17 cells secrete inflammatory factors IL-17 and IL-23 through the specific transcription regulator ROR*γ*t, thereby aggravating inflammatory responses, and Treg cells can be converted into Th17 cells in the presence of IL-6 and IL-23, resulting in an imbalance of the Treg/Th17 immune axis [45–48]. Treg cells play an immune regulatory role by secreting inflammatory inhibitors, such as IL-10 and TGF-*β* through the transcription regulator Foxp3 [45–48]. Therefore, we determined the proportion of Th17 cells and Treg cells and the related factors. These results showed that after treatment, the proportion of Th17 cells was significantly decreased, and the proportion of Treg cells was significantly increased, the serum levels of the inflammatory cytokines IL-6 and IL-17 were significantly reduced, the levels of anti-inflammatory cytokines IL-10 and TGF-*β*1 were significantly increased, especially in the SSW-H and mesalazine groups.

STAT3, which plays a key role in maintaining the intestinal mucosal barrier, is an important transcription factor that causes the onset of colitis. STAT3 is activated and phosphorylated by IL-6 and thus participates in the inflammatory response [49, 50]. STAT3, as one of the important factors affecting UC, promotes the inflammation and pathogenesis of UC, and its overexpression inhibits the production of anti-inflammatory factors [51]. p-STAT3 inhibits the differentiation of CD4^+^T cells into Treg cells and promotes their differentiation into Th17 cells, leading to an imbalance of the Treg/Th17 immune axis [6, 7, 52, 53]. In previous studies, it has been shown that PPAR*γ* is highly expressed in the colon, and activation of PPAR*γ* has a protective effect on colitis, while its expression is reduced in patients with UC [54–58]. Activation of PPAR*γ* reduces local intestinal IL-6 production and inhibits the elimination of coreceptor SMRT by ROR*γ*t promoter, thereby inhibiting the ROR*γ*t-induced differentiation of Th17 cells [55, 58–60]. Therefore, we determined the related proteins that affect the imbalance of the Treg/Th17 immune axis. These results showed that after treatment, the expression of TGF-*β*1 and PPAR*γ* in colon tissue of rats in all treatment groups was significantly increased, and the expression of STAT3 and p-STAT3 was significantly decreased, especially in rats in the SSW-H and mesalazine groups.

SSW can correct the imbalance of Treg/Th17 in UC rats, but whether it improves the imbalance by regulating the intestinal microbiota is still unclear. We consulted the relevant literature and found that the occurrence of UC is accompanied by intestinal microbiota dysbiosis, which changes the diversity and abundance of gut microbiota, thereby increasing Bacteroidota and decreasing Firmicutes [61–63]. In previous studies, it has been shown that the gut microbiota of IBD patients can affect the balance of Th17 and ROR*γ*t+ Treg cells in the intestinal tract of mice [64]. The expression of PPAR*γ* in intestinal epithelial cells is closely related to the gut microbiota and can activate the expression of PPAR*γ* in the intestinal tract and maintain the intestinal mucosal homeostasis [58]. SCFAs are a type of anti-inflammatory bacterial metabolites that regulate the differentiation direction of CD4^+^T cells, restore the immune axis balance, and inhibit inflammatory responses [65]. As one of the main components of SCFAs, butyrate is a key energy source for colonic and intestinal cells, and can reduce the intestinal permeability, enhance the intestinal mucosal barrier, down-regulate the expression of proinflammatory cytokines IL-6, and activate PPAR*γ*, thereby reducing the severity of UC [66–68]. Human colonic butyric acid-producing bacteria belong to Gram-positive firmicutes [69–72]. Therefore, we determined the content of butyric acid and the correlation analysis between butyric acid production and various phylum and genus. The results showed that the concentration of butyric acid increased in all treatment groups, especially in the SSW-H and mesalazine group. The results of the correlation analysis between gut microbiota and SCFAs showed that at the phylum level, butyric acid had a positive correlation with Firmicutes and a negative correlation with Bacteroidota. Furthermore, at the genus level, there was a positive correlation between butyric acid and *Lactobacillus*. The relative abundance of intestinal microbiota in rat feces was determined. The results obtained in this study showed that the relative abundance of Firmicutes was increased and that of Bacteroidota was decreased in all treatment groups when analyzed at the phylum level. At the genus level, the relative abundance of *Lactobacillus* increased. In summary, SSW may regulate the relative abundance of Firmicutes and the butyric acid concentration in the gut microbiota and regulate the balance of the Treg/Th17 immune axis, thereby restoring intestinal homeostasis to treat UC.

## 5. Conclusion

In this study, it was found that SSW could correct the imbalance of the Treg/Th17 immune axis in colon tissue of a DNBS-induced UC rat model and played a role in reducing inflammatory responses and tissue damage. SSW can increase the concentration of butyric acid in the gut microbiota, increase the expression of PPAR*γ*, inhibit the production of inflammatory cytokines, and improve damaged intestinal barrier function under the inflammatory state, thereby correcting the imbalance of the Treg/Th17 immune axis and reducing inflammatory responses and tissue damage. Therefore, our findings showed that SSW can regulate the gut microbiota, restore immune function, and provide a potentially effective treatment for UC.

## Figures and Tables

**Table 1:** Effect of modeling on the rat body weight.

Group	Body weight on day 0 (g)	Body weight on day 11 (g)	Body weight on day 21 (g)
Blank	200.5 ± 10.9	231.3 ± 9.8	242.9 ± 12.1
Model	199.6 ± 13.3	202.3 ± 10.7	205.8 ± 12.0

**Table 2:** Effect of SSW on the body weight of UC rats.

Group	Body weight on day 0 of drug intervention (g)	Body weight on day 21 of drug intervention (g)
Blank	242.9 ± 12.1	264.6 ± 9.0
Model	205.8 ± 12.0	230.9 ± 6.0^*∗∗*^
SSW-L	207.1 ± 15.2	233.9 ± 7.3^*∗∗*^^◆◆^
SSW-M	203.6 ± 14.9	240.6 ± 8.8^*∗∗*^^#◆^
SSW-H	205.6 ± 14.6	249.1 ± 9.3^*∗∗*^^##^
Mesalazine	209.1 ± 16.4	251.9 ± 10.7^*∗∗*^^##^

**Table 3:** Effect of SSW on the DAI Score of UC rats.

Group	DAI score at the end of modeling (point)	DAI score at the end of drug intervention (point)
Blank	0.00 ± 0.00	0.00 ± 0.00
Model	2.67 ± 0.18^*∗∗*^	2.58 ± 0.15^*∗∗*^
SSW-L	—	1.96 ± 0.12^*∗∗*^^##◆◆^
SSW-M	—	1.71 ± 0.28^*∗∗*^^##◆◆^
SSW-H	—	1.00 ± 0.18^*∗∗*^^##^
Mesalazine	—	0.96 ± 0.12^*∗∗*^^##^

^*∗∗*^ *P* < 0.01 compared with the blank group; ^##^*P* < 0.01, ^#^*P* < 0.05 compared with the model group; ^◆◆^*P* < 0.01, ^◆^*P* < 0.05 compared with mesalazine group (mean ± SD, *n* = 8).

**Table 4:** Effect of SSW on the CMDI score of colonic mucosa in UC rats.

Group	CMDI score (points)
Blank	0
Model	3.88 ± 0.35^*∗∗*^
SSW-L	2.88 ± 0.64^*∗∗*^^##◆◆^
SSW-M	2.38 ± 0.52^*∗∗*^^##◆^
SSW-H	1.38 ± 0.52^*∗∗*^^##^
Mesalazine	1.25 ± 0.46^*∗∗*^^##^

^*∗∗*^ *P* < 0.01 compared with the blank group; ^##^*P* < 0.01 compared with the model group; ^◆◆^*P* < 0.01, ^◆^*P* < 0.05 compared with the mesalazine group (mean ± SD, *n* = 8).

**Table 5:** Effect of SSW on the HS score of colon tissue in UC rats.

Group	HS score (points)
Blank	0
Model	3.86 ± 0.35^*∗∗*^
SSW-L	3.00 ± 0.53^*∗∗*^^##◆◆^
SSW-M	2.25 ± 0.46^*∗∗*^^##◆◆^
SSW-H	1.25 ± 0.46^*∗∗*^^##^
Mesalazine	1.13 ± 0.35^*∗∗*^^##^

^*∗∗*^ *P* < 0.01 compared with the blank group; ^##^*P* < 0.01 compared with the model group; ^◆◆^*P* < 0.01 compared with the mesalazine group (mean ± SD, *n* = 8).

**Table 6:** Effects of SSW on the levels of cytokines in colon tissues of UC rats.

Group	IL-6	IL-17	IL-10	TGF-*β*1
Blank	34.46 ± 13.54	6.92 ± 2.22	258.21 ± 44.35	380.23 ± 24.27
Model	242.89 ± 15.17^*∗∗*^	30.85 ± 3.56^*∗∗*^	34.69 ± 14.72^*∗∗*^	162.25 ± 13.32^*∗∗*^
SSW-L	200.19 ± 13.00^*∗∗*^^##◆◆^	22.29 ± 3.92^*∗∗*^^##◆◆^	58.13 ± 15.67^*∗∗*^^◆◆^	232.79 ± 11.48^*∗∗*^^##◆◆^
SSW-M	159.05 ± 10.02^*∗∗*^^##◆◆^	18.73 ± 3.63^*∗∗*^^##◆^	91.29 ± 8.52^*∗∗*^^#◆^	256.15 ± 11.41^*∗∗*^^##◆◆^
SSW-H	99.91 ± 18.88^*∗∗*^^##^	13.32 ± 2.68^*∗*^^##^	138.20 ± 30.98^*∗∗*^^##^	308.09 ± 9.37^*∗∗*^^##^
Mesalazine	82.06 ± 26.69^*∗∗*^^##^	11.57 ± 4.05^*∗*^^##^	151.91 ± 46.59^*∗∗*^^##^	326.21 ± 18.58^*∗∗*^^##^

**Table 7:** Effects of SSW on the relative abundance of gut microbiota at the phylum level in UC rats.

Group	Firmicutes (relative abundance)	Bacteroidota (relative abundance)
Blank	0.70 ± 0.10	0.28 ± 0.09
Model	0.56 ± 0.07^*∗∗*^	0.40 ± 0.05^*∗*^
SSW-L	0.57 ± 0.06^*∗*^	0.31 ± 0.07
SSW-M	0.65 ± 0.09	0.25 ± 0.07^##^
SSW-H	0.73 ± 0.03^##^	0.20 ± 0.04^##^
Mesalazine	0.71 ± 0.11^##^	0.23 ± 0.11^##^

**Table 8:** Effects of SSW on the relative abundance of gut microbiota at the genus level in UC rats.

Group	*Lactobacillus*(relative abundance)
Blank	0.32 ± 0.07
Model	0.06 ± 0.05^*∗∗*^
SSW-L	0.13 ± 0.04^*∗∗*^^#◆◆^
SSW-M	0.18 ± 0.03^##◆◆^
SSW-H	0.26 ± 0.04^##^
Mesalazine	0.29 ± 0.05^##^

^*∗∗*^ *P* < 0.01, ^*∗*^*P* < 0.05 compared with the blank group; ^##^*P* < 0.01, ^#^*P* < 0.05 compared with the model group; ^◆◆^*P* < 0.01 compared with the mesalazine group (mean ± SD, *n* = 6).

**Table 9:** Effect of SSW on the butyric acid concentration in UC rats.

Group	Butyric acid (*μ*g/g)
Blank	903.51 ± 167.24
Model	401.14 ± 191.77^*∗∗*^
SSW-L	539.92 ± 215.79^*∗∗*^
SSW-M	610.10 ± 191.92^*∗*^
SSW-H	633.66 ± 139.05^*∗*^^#^
Mesalazine	663.07 ± 217.72^*∗*^^#^

^*∗∗*^ *P*  <  0.01, ^*∗*^*P* < 0.05 compared with the blank group; ^#^*P* < 0.05 compared with the model group (mean ± SD, *n* = 6).

## Data Availability

All the data related to this article are described as pictures and statistical analysis in the manuscript.
